# Supplementary material for: Clonal reversal of ageing-associated stem cell lineage bias via a pluripotent intermediate
Source: Nat Commun. 2017 Feb 22;8:14533. doi: 10.1038/ncomms14533 (PMC5322498; doi:10.1038/ncomms14533)
Supplement: Supplementary Information — Supplementary Figures and Supplementary Tables [file ncomms14533-s1.pdf]

SUPPLEMENTARY INFORMATION

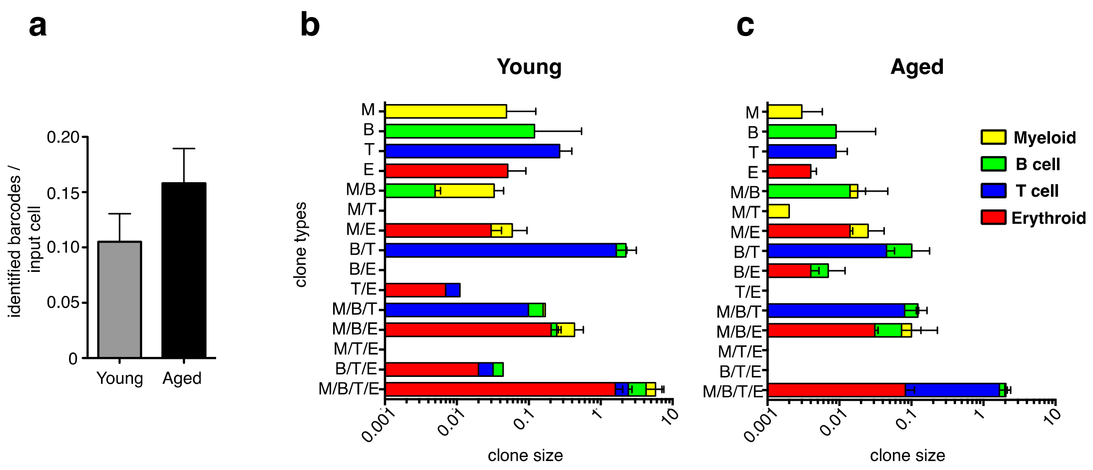

Supplementary Figure 1. A majority of HSC clonal subtypes display lower output of mature effector cells with age. (**a**) Bar chart depicting the overall barcode detection in recipients transplanted with young and aged barcoded HSCs expressed as barcodes / input HSCs (= transduced and transplanted HSCs, n = 5 mice per group). The clone sizes of all young (**b**) and aged (**c**) mono-, bi- and multipotent HSC clones were determined. The data is from one experiment. Error bars indicate mean ± SD.

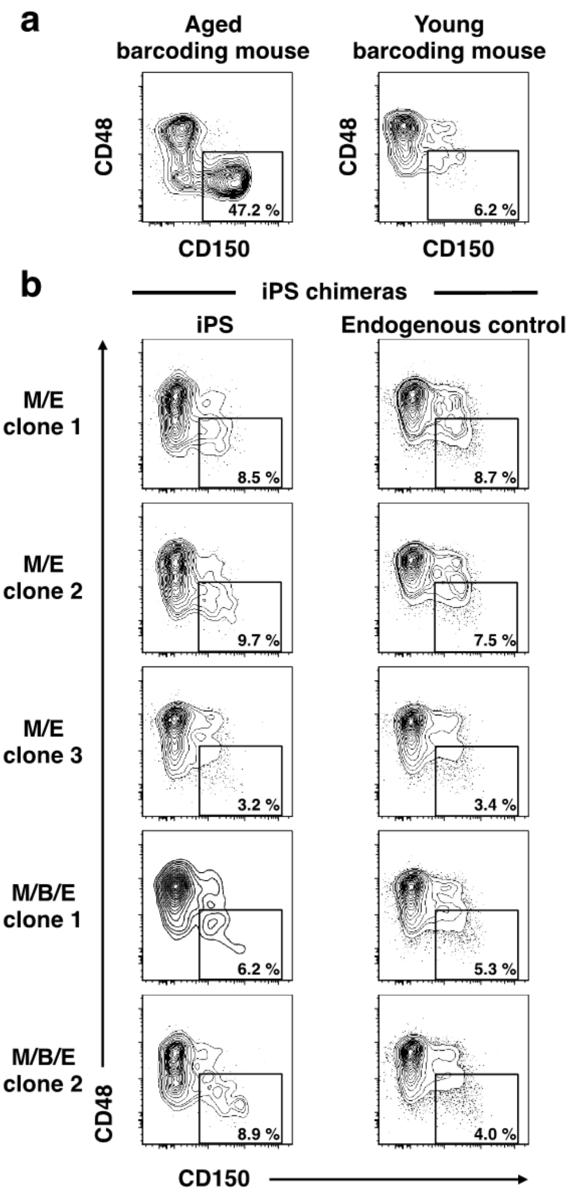

Supplementary Figure 2. IPS-HSCs express CD150 at levels comparable to those in young mice. (**a**) FACS plots depicting the CD48 and CD150 expression profile and the frequency of $CD48^{-}CD150^{+}$ HSCs of the aged (LEFT) and young (RIGHT) HSCs used for barcoding (pregated on single, viable, $lineage^{-}$, $c\text{-}Kit^{+}$, $Sca\text{-}1^{+}$ cells), (**b)** and of the iPS (LEFT) and endogenous control (RIGHT) hematopoiesis of each of the iPS chimeras (pregated on single, viable, $lineage^{-}$, $c\text{-}Kit^{+}$, $Sca\text{-}1^{+}$, $CD45.2^{+}$ or $CD45.1^{+}/.2^{+}$ cells for iPS and endogenous respectively). The data is from three experiments.

a

FACS gating strategy for analysis and cell isolation from the peripheral blood of 1° transplants (young cells transplanted)

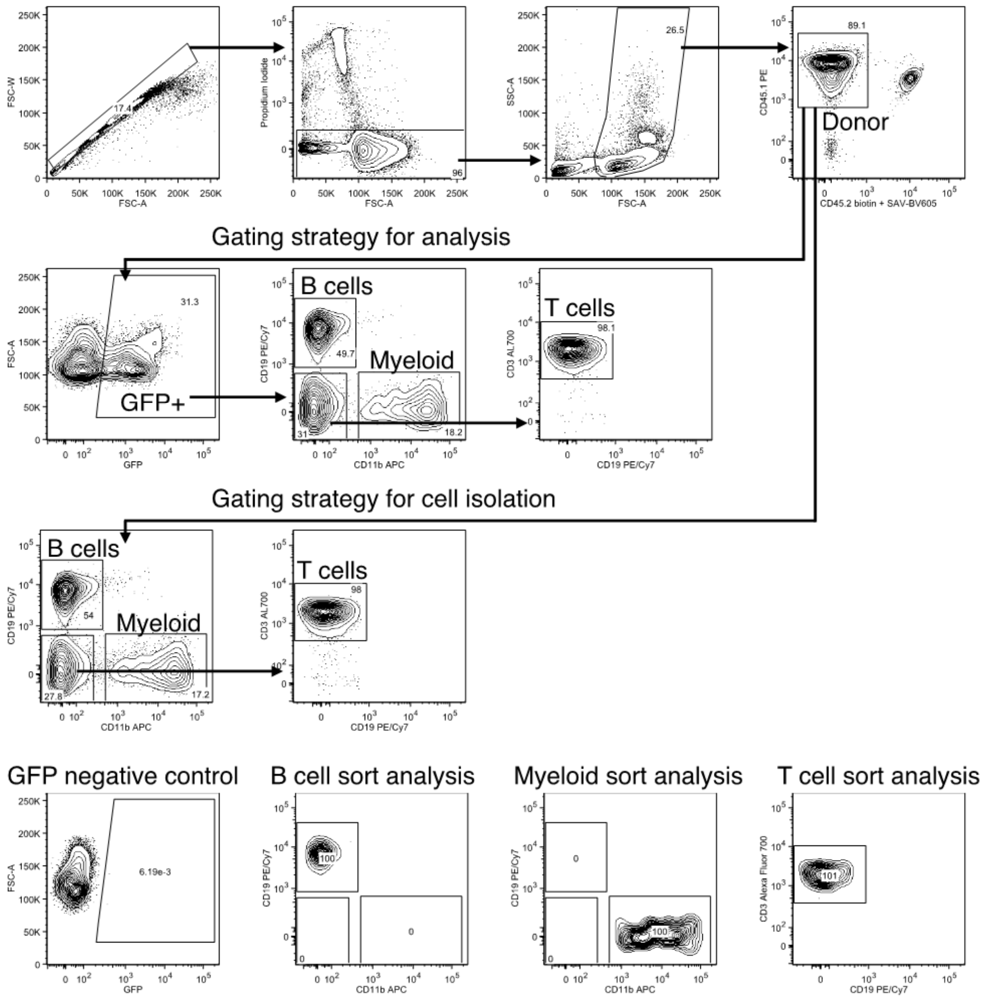

b

FACS gating strategy for analysis and cell isolation from the peripheral blood of 1° transplants (aged cells transplanted)

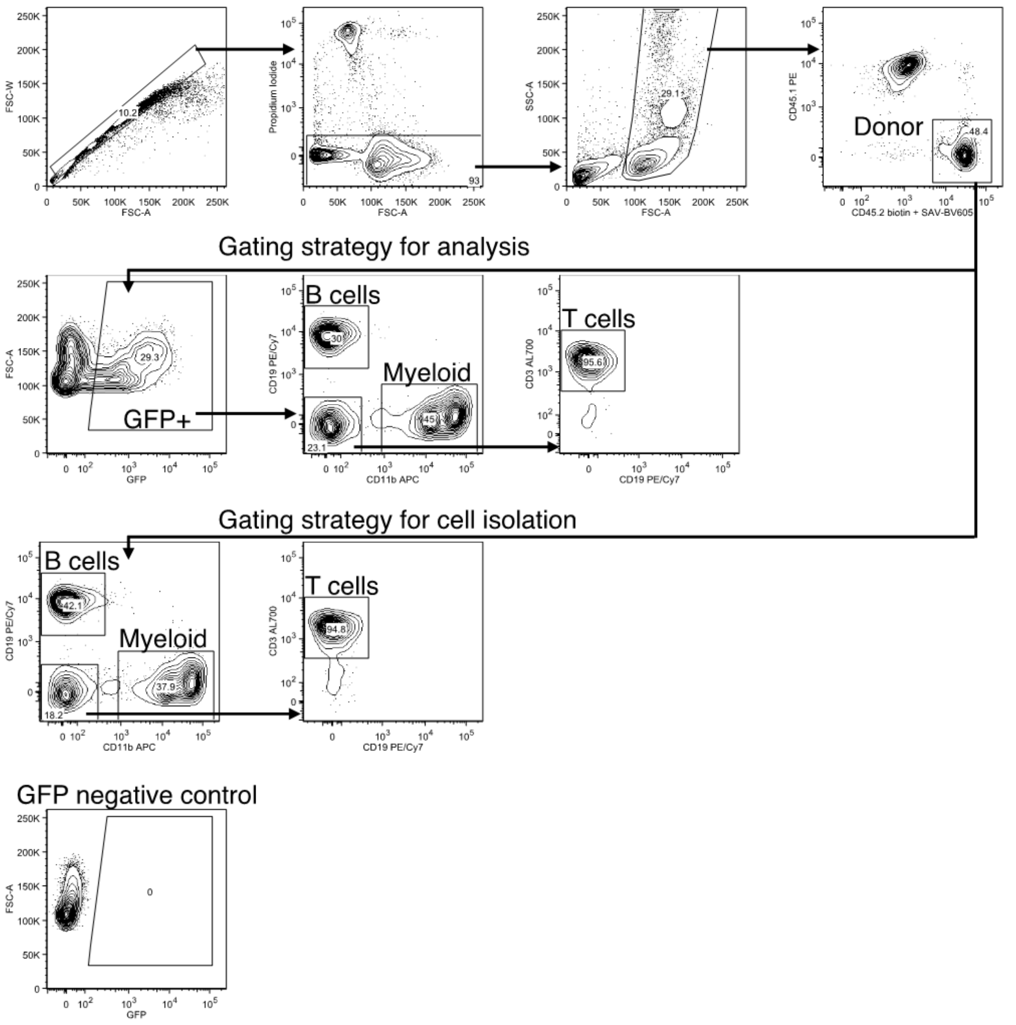

c

FACS gating strategy for cell isolation from the BM of 1° transplants (young cells transplanted)

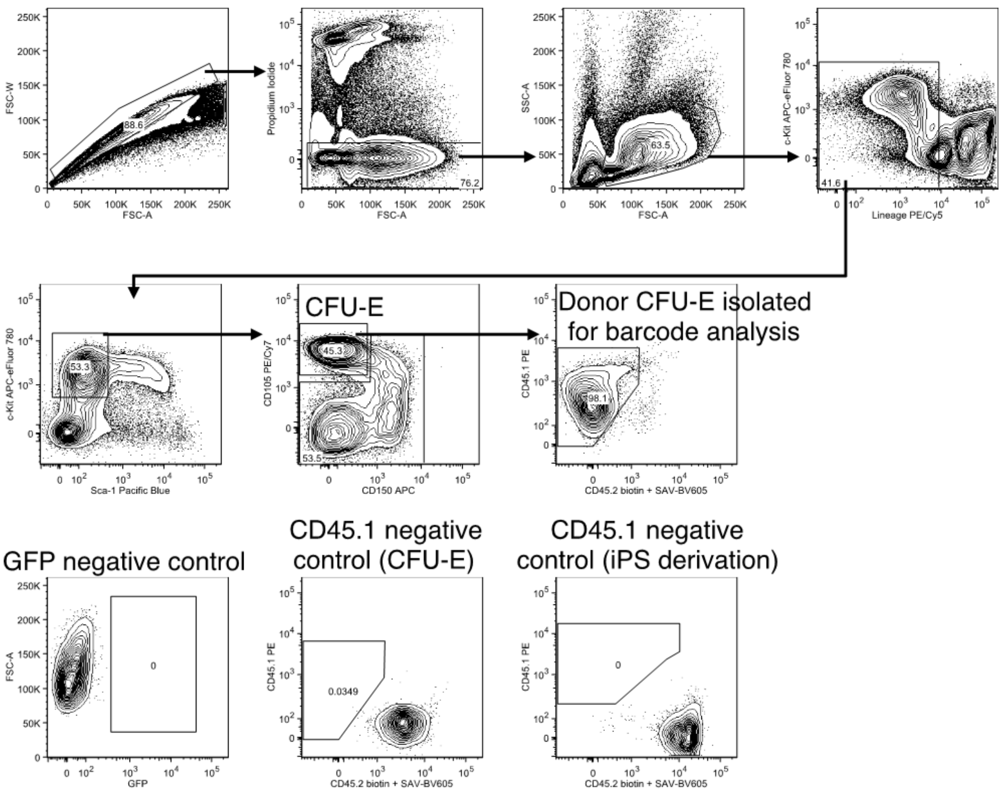

d

FACS gating strategy for cell isolation from the BM of 1° transplants (aged cells transplanted)

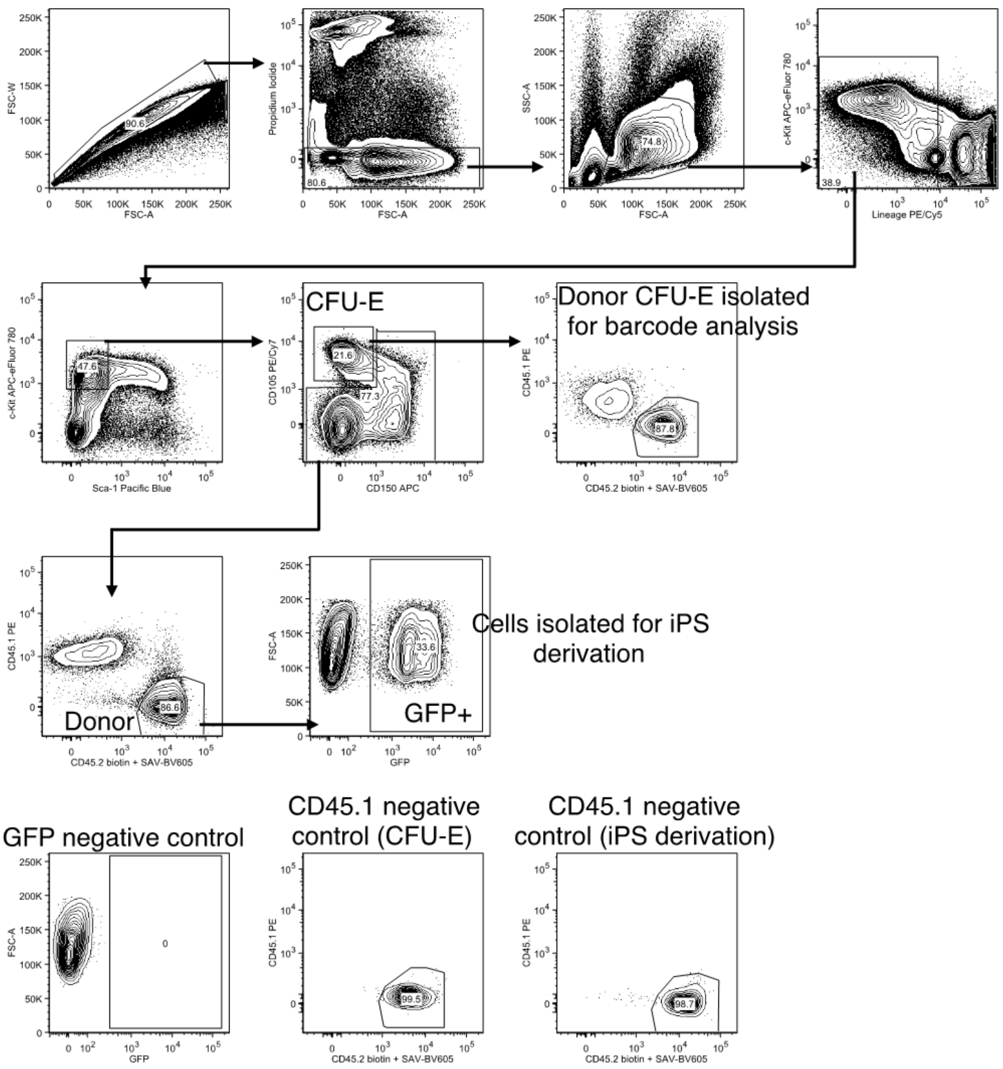

e

FACS gating strategy for HSC analysis and cell isolation from the BM of iPS chimeras and 2° transplants

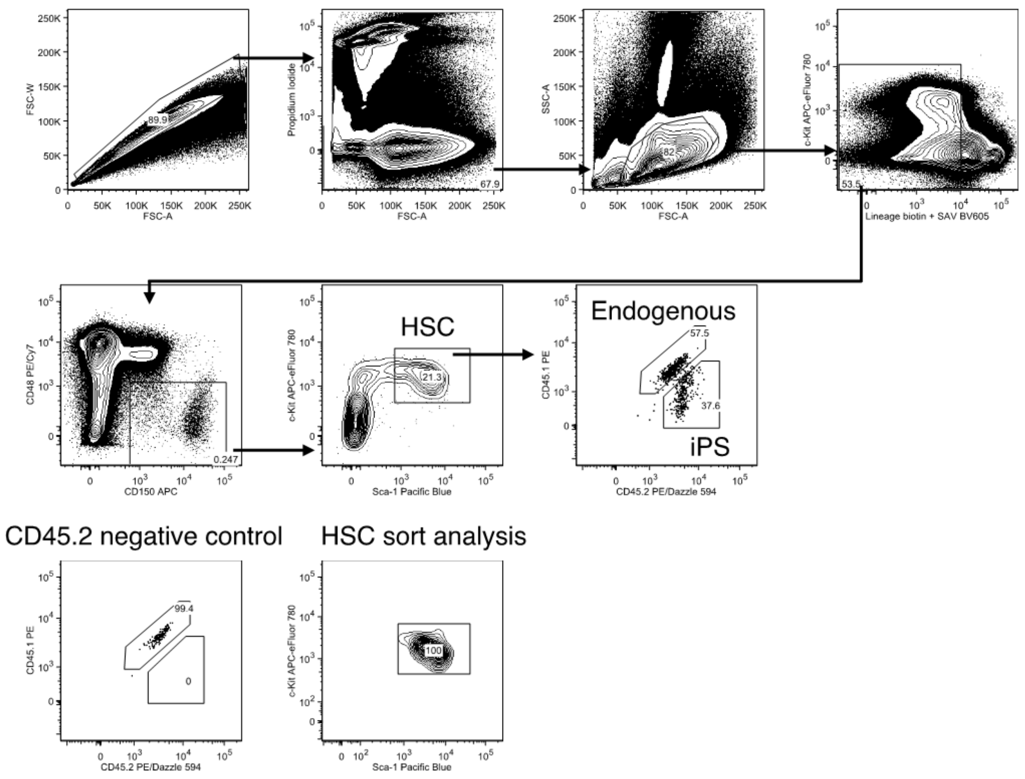

f

FACS gating strategy for peripheral blood analysis in iPS chimeras and 2° transplants

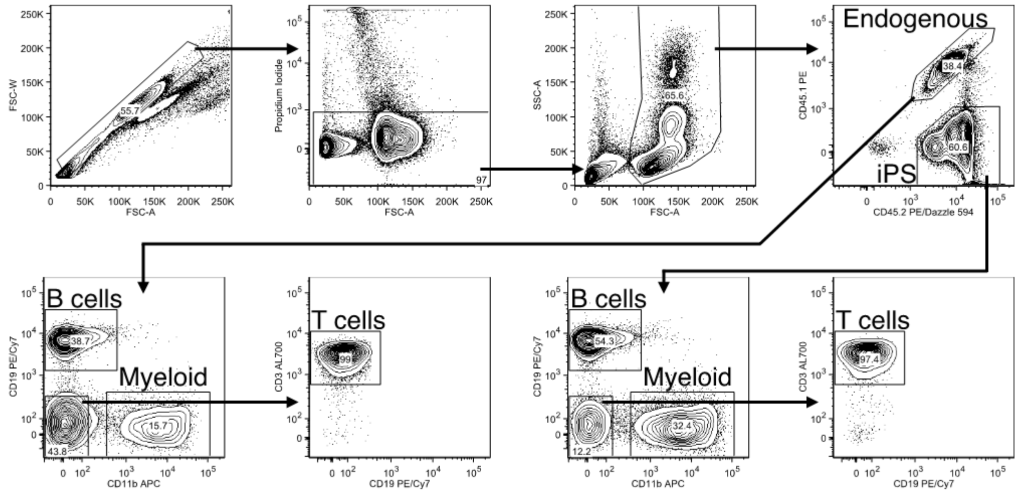

g

FACS gating strategy for analysis of thymi in iPS chimeras and 2° transplants

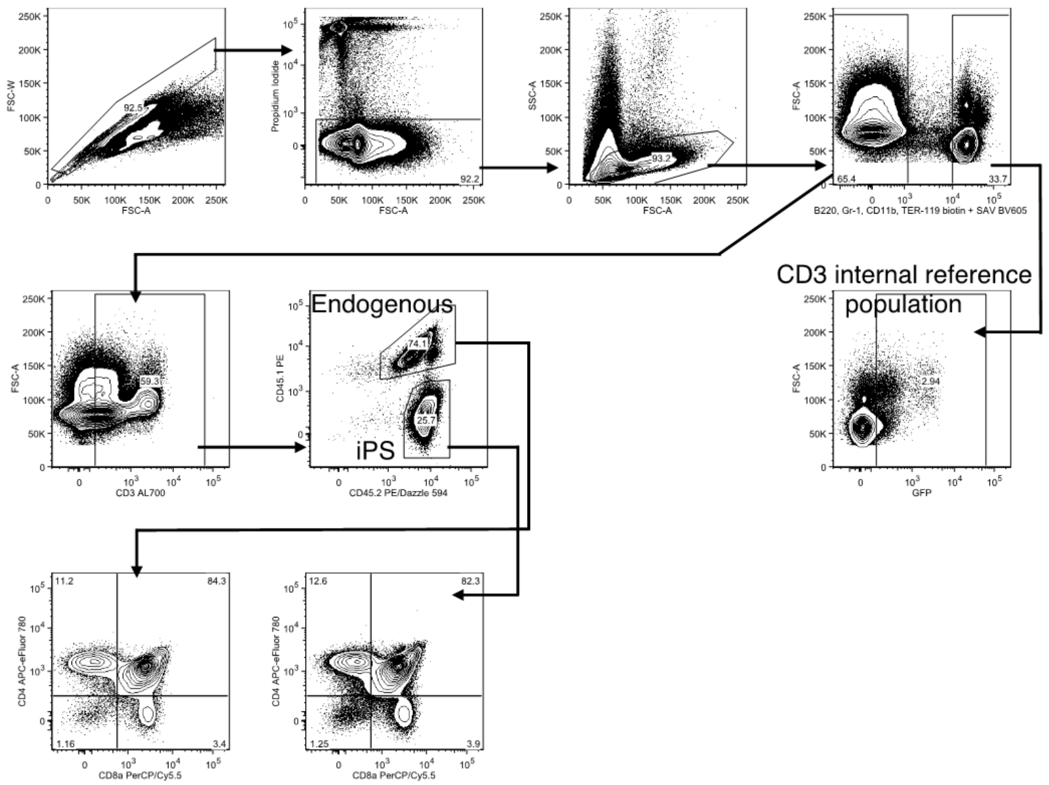

Supplementary Figure 3. Example FACS plots (a-g).

Gene symbol	Fluidigm Assay ID	Fluidigm Delta Gene Assay Name	Forward primer	Reverse primer
Actb	GEA00011830	Actb_11830_i2	CCCTAAGGCCAACCGTGAAA	CAGCCTGGATGGCTACGTAC
Alcam	GEA00013337	Alcam_13337_i11	GCAGAAAACCAACTGGAGAGAAC	GCCTCATCGTGCTCTGGAATA
Aldh1a1	GEA00012002	Aldh1a1_12002_i10	AGACCTGGATAAGGCCATCA	CACTGGGCTGACAACATCA
Arhgap30	in-house design	in-house design	GAGCATCTACAGCACTCGGG	AGATCCCATCCACCACTCCA
Bmpr1a	in-house design	in-house design	TCTGGGAGTGGATCTGGATT	ATAGCGGCCTTTACCAACCT
Camk1d	in-house design	in-house design	AGAACGAGATTGCCGTGCTT	CCAGGTAGAGGTGATTTGGGC
Cited2	in-house design	in-house design	CTGCCGCCCAATGTCATAGA	CTCCTTGATGCGGTCCAAAC
Clca1	in-house design	in-house design	ACAGTGTGGGGACAGAGGAC	GCCCACTCATGGACAAAGAC
Clec1A	in-house design	in-house design	CCTTGTGTTTGGTGCTGCTC	AGTGATGCTGTCTTGCTGGA
Clu	GEA00043739	Clu_43739_i5	AACTCCACAGGATGCCTGAA	GGGCAGGATTGTTGGTTGAA
Ebi3	in-house design	in-house design	CATCTCCTGCCCCATACACT	CCCATCAGAGAGAGGGTGAA
Ezh2	GEA00013271	Ezh2_13271_i4	TGATGGAAAAGTGCATGGTGAC	GACCAAGAGCATTTACCAACTCC
Fap	in-house design	in-house design	CCTTGCATCTGGAACTGGTC	GAGGCCCATGAATCTCTCTG
Fhl1	in-house design	in-house design	GTGGCCAAGAAGTGTGCTG	TGCAGTGGAAGCAGTAGTCG
Gadd45g	GEA00013398	Gadd45g_13398_i0	AGAAGTCCGTGGCCAGGATA	GAAGTTCGTGCAGTGCTTTCC
Gapdh	GEA00007851	Gapdh_7851_i0	AGACGGCCGCATCTTCTT	TTCACACCGACCTTCACCAT
Gata1	GEA00012861	Gata1_12861_i1	GCATCAGCACTGGCCTACTA	TTGAGCAGTGGATACACCTGAA
Gda	in-house design	in-house design	AAGGTTCCGGAGCACTGATG	CGCAAGGATCAGGGATGAGT
Ghr	in-house design	in-house design	CTGCAAAGAATCAATCCAAGCC	CAGTTCAGGGGAACGACACTT
Gm106	in-house design	in-house design	GTGTGAATGGGCCGAGTATT	AAGCGTAGCAATGCGAGAGT
Hlf	GEA00038068	Hlf_38068_i2	TGAAGCCACAGCCCATGATTA	CCTCGCCCAGTACTTGTCA
Hmga2	in-house design	in-house design	CACTTGTCAGCCTTGAAGCA	GCTCCTCCCATGTCATCACT
Hnf4a	in-house design	in-house design	TATGCCTGCCTCAAAGCCAT	ACTGCCGGTCGTTGATGTAA
Hprt	GEA00002682	Hprt_2682_i5	CAGTACAGCCCCAAAATGGTTA	AGTCTGGCCTGTATCCAACA
Id2	GEP00055173	Id2_55173_i0	ACCCTGAACACGGACATCA	TCGACATAAGCTCAGAAGGGAA
Itgb3	in-house design	in-house design	GTCACATTGGCACCGACAAC	TCTCAGTCATCAGCCCCAGA
Jakmip1	in-house design	in-house design	GGAGTGCGAACGTGACATC	GTTTGCACACCAAGCTCCTT
Jam2	GEA00044742	Jam2_44742_i4	CACAACAACAGCTCGTACACA	CTCTCCACTGTCCATCTTGGAA

Lpl	in-house design	in-house design	GCCCAGCAACATTATCCAGT	TGGACGTTGTCTAGGGGGTA
Meis1	GEA00017910	Meis1_17910_i8	AGTTGGCACAAGATACAGGAC	GGGCTGCACTATTCTTCTCC
Mmp2	in-house design	in-house design	ATGACATCAAGGGGATCCAG	GGAGTGACAGGTCCCAGTGT
Neo1	in-house design	in-house design	GCCCAGACCATCAAAGTGGA	GGATGCCTTCGCCAACATTG
Nupr1	GEA00037007	Nupr1_37007_i0	CCCTTCCCAGCAACCTCTAA	TCTCTTGGTCCGACCTTTCC
Osmr	GEA00022618	Osmr_22618_i15	GCTCAGCATCATTGTCTGCTAC	TGCTCTTGTACGGATTGGGAA
Pgr	in-house design	in-house design	ACCCGCCATACCTTAACTACC	CGCCATAGTGACAGCCAGAT
Plac8	in-house design	in-house design	GCTCAGGCACCAACAGTTATC	GCTGCCACTTGACATCCAAGA
Plscr2	in-house design	in-house design	GTATCCTCCAGCAGCAGTCC	GCCGTGTAGCAGCATAACCT
Rassf4	in-house design	in-house design	GGTGCTGGACAGTTTTGTTG	GACGCTGCAGCATTGTTAGA
Rnase6	in-house design	in-house design	CTGTGGGAGCCGATGTATCT	GTTGCATGGTTGACGACTTG
Runx1t1	in-house design	in-house design	AGTTCGCACCCTTGTACTGG	AAACGGGATGACAAAAGGTCT
Satb1	in-house design	in-house design	AACACTCGGGCCATCTCATG	TACAAATTCCGCGTGCTCCT
Sdpr	GEA00022586	Sdpr_22586_i0	TCCGACGCAACCACTTCAAA	ACACACTGGCAGGGATCTCA
Selp	GEA00022641	Selp_22641_i13	ATGGAAAATGCCCCTTGAACC	AAGGGGTTGGGTCATATGCA
Slamf1	GEA00011782	Slamf1_11782_i4	GCCAGCCACCAGTGGAA	GTCCTGATCTGTTAGGGCATCA
Socs3	in-house design	in-house design	AAGGGAGGCAGATCAACAGA	GCCCCACATAGGAGAGACAA
Sult1a1	in-house design	in-house design	CCAGCCCCACGGATCATTAAG	CGGGCAACGTAGATCACCTTG
Vwf	GEA00013394	vwf_13394_i29	AGTTTGGTGGACCTCATGCA	GTTACATAGCGCACGGCAAA
Wwtr1	in-house design	in-house design	CTGCCATGAGCACAGATATGA	TAACCCCAGGCCACTGTCT

Supplementary Table 1. Description of the gene expression assays used for the Fluidigm multiplexed quantitative RT-PCR experiments.

Antigen	Conjugate	Antibody clone	Supplier	Concentration	Dilution factor
B220	Biotin	RA3-6B2	Biolegend	0.5 mg/ml	1:400
B220	PE/Cy5	RA3-6B2	Biolegend	0.2 mg/ml	1:400
CD105	PE/Cy7	MJ7/18	Biolegend	0.2 mg/ml	1:200
CD117/Kit	APC-eFluor 780	2B8	eBioscience	0.2 mg/ml	1:200
CD11b	Biotin	M1/70	Biolegend	0.5 mg/ml	1:400
CD11b	APC	M1/70	Biolegend	0.2 mg/ml	1:800
CD11b	PE/Cy5	M1/70	Biolegend	0.2 mg/ml	1:400
CD150	APC	TC15-12F12.2	Biolegend	0.2 mg/ml	1:400
CD19	PE/Cy7	1D3	eBioscience	0.2 mg/ml	1:400
CD3	Alexa Fluor 700	17A2	Biolegend	0.5 mg/ml	1:400
CD3	PE/Cy5	145-2C11	Biolegend	0.2 mg/ml	1:400
CD4	Biotin	RM4-5	Biolegend	0.5 mg/ml	1:400
CD4	APC-eFluor 780	RM4-5	eBioscience	0.2 mg/ml	1:200
CD4	Brilliant Violet 711	RM4-5	Biolegend	0.2 mg/ml	1:200
CD45.1	PE	A20	Biolegend	0.2 mg/ml	1:200
CD45.2	Biotin	104	Biolegend	0.5 mg/ml	1:200
CD45.2	PE/Dazzle 594	104	Biolegend	0.2 mg/ml	1:200
CD48	FITC	HM48-1	Biolegend	0.5 mg/ml	1:200
CD48	Alexa Fluor 700	HM48-1	Biolegend	0.5 mg/ml	1:200
CD48	PE/Cy7	HM48-1	Biolegend	0.2 mg/ml	1:200
CD8a	Biotin	53-6.7	Biolegend	0.5 mg/ml	1:400
CD8a	PerCP/Cy5.5	53-6.7	Sony Biotechnology	0.2 mg/ml	1:200
Gr-1	Biotin	RB6-8C5	Biolegend	0.5 mg/ml	1:400
Gr-1	PE/Cy5	RB6-8C5	Biolegend	0.2 mg/ml	1:400
Sav	PE/Cy5		eBioscience	0.2 mg/ml	1:400
Sav	Brilliant Violet 605		Biolegend	0.1 mg/ml	1:400
Sca1	Pacific Blue	E13-161.7	Biolegend	0.5 mg/ml	1:200
TER-119	Biotin	TER-119	Biolegend	0.5 mg/ml	1:400
TER-119	PE/Cy5	TER-119	Biolegend	0.2 mg/ml	1:400

Supplementary Table 2. Antibodies used for FACS experiments.

	Young #1	Young #2	Young #3	Young #4	Young #5	Old #1	Old #2	Old #3	Old #4	Old #5
**Myeloid cells, raw reads**	63092	71863	84635	84667	66058	93761	99325	98484	72100	99579
**Myeloid cells, quality- and length filtered BC reads**	27467	30305	29577	24323	30021	45617	31942	40927	36792	44000
**Corresponding to # of BCs**	226	341	173	279	152	728	798	814	526	892
**# of barcodes following Starcode consolidation**	30	25	21	36	25	218	324	287	225	280
**# of BCs filtered by clone size cut-off and used for downstream analyses**	13 (27)	9 (51)	9 (11)	17 (12)	12 (54)	146 (8)	229 (5)	207 (8)	171 (7)	194 (8)
**(cut-off in # reads to call BCs)**										
**B cells, raw reads**	90719	104498	100749	175094	134909	84611	121128	127109	95514	91804
**B cells, quality- and length filtered BC reads**	53672	74331	51273	110039	109271	54177	41059	61745	55078	63756
**Corresponding to # of BCs**	294	355	324	452	174	461	753	520	552	548
**# of barcodes following Starcode consolidation**	70	49	52	70	27	215	401	249	309	286
**# of BCs filtered by clone size cut-off and used for downstream analyses**	35 (18)	19 (34)	27 (5)	21 (70)	13 (95)	158 (3)	232 (9)	152 (11)	174 (12)	183 (11)
**(cut-off in # reads to call BCs)**										
**T cells, raw reads**	91280	99565	97209	108619	118254	0	117824	119947	142800	112377
**T cells, quality- and length filtered BC reads**	46648	51148	49043	53887	73172	0	67362	43277	28460	88266
**Corresponding to # of BCs**	239	433	391	373	268	0	205	109	114	147
**# of barcodes following Starcode consolidation**	30	38	33	48	27	0	41	15	20	21
**# of BCs filtered by clone size cut-off and used for downstream analyses**	13 (19)	15 (143)	14 (5)	16 (54)	12 (49)	0	30 (2)	10 (2)	10 (8)	8 (74)
**(cut-off in # reads to call BCs)**										
**Ery progenitors, raw reads**	87630	68339	105442	153315	104162	122867	125528	138007	118465	131394
**Ery progenitors, quality- and length filtered BC reads**	45473	39228	37052	71574	68056	82714	57026	74770	74561	80215
**Corresponding to # of BCs**	258	205	194	417	164	618	629	641	582	648
**# of barcodes following Starcode consolidation**	33	15	29	51	23	204	219	222	193	235
**# of BCs filtered by clone size cut-off and used for downstream analyses**	13 (23)	7 (33)	10 (33)	13 (61)	14 (122)	127 (13)	140 (10)	153 (15)	129 (15)	160 (14)
**(cut-off in # reads to call BCs)**										

Supplementary Table 3. Sequencing coverage and barcode detection/filtration details.
